# The Proton Pump Inhibitor Omeprazole Does Not Promote Clostridioides difficile Colonization in a Murine Model

**DOI:** 10.1128/mSphere.00693-19

**Published:** 2019-11-20

**Authors:** Sarah Tomkovich, Nicholas A. Lesniak, Yuan Li, Lucas Bishop, Madison J. Fitzgerald, Patrick D. Schloss

**Affiliations:** a Department of Microbiology and Immunology, University of Michigan, Ann Arbor, Michigan, USA; Antimicrobial Development Specialists, LLC

**Keywords:** 16S rRNA gene, *Clostridioides difficile*, *Clostridium difficile*, colonization resistance, infection, microbial ecology, microbiome, mouse model, pathogenesis

## Abstract

Proton pump inhibitor (PPI) use has been associated with microbiota alterations and susceptibility to Clostridioides difficile infections (CDIs) in humans. We assessed how PPI treatment alters the fecal microbiota and whether treatment promotes CDIs in a mouse model. Mice receiving a PPI treatment were gavaged with 40 mg of omeprazole per kg of body weight during a 7-day pretreatment phase, the day of C. difficile challenge, and the following 9 days. We found that mice treated with omeprazole were not colonized by C. difficile. When omeprazole treatment was combined with a single clindamycin treatment, one cage of mice remained resistant to C. difficile colonization, while the other cage was colonized. Treating mice with only clindamycin followed by challenge resulted in C. difficile colonization. 16S rRNA gene sequencing analysis revealed that omeprazole had minimal impact on the structure of the murine microbiota throughout the 16 days of omeprazole exposure. These results suggest that omeprazole treatment alone is not sufficient to disrupt microbiota resistance to C. difficile infection in mice that are normally resistant in the absence of antibiotic treatment.

**IMPORTANCE** Antibiotics are the primary risk factor for Clostridioides difficile infections (CDIs), but other factors may also increase a person’s risk. In epidemiological studies, proton pump inhibitor (PPI) use has been associated with CDI incidence and recurrence. PPIs have also been associated with alterations in the human intestinal microbiota in observational and interventional studies. We evaluated the effects of the PPI omeprazole on the structure of the murine intestinal microbiota and its ability to disrupt colonization resistance to C. difficile. We found omeprazole treatment had minimal impact on the murine fecal microbiota and did not promote C. difficile colonization. Further studies are needed to determine whether other factors contribute to the association between PPIs and CDIs seen in humans or whether aspects of murine physiology may limit its utility to test these types of hypotheses.

## Abstract (precis)

Antibiotics are the primary risk factor for Clostridioides difficile infections (CDIs), but other factors may also increase a person’s risk. In epidemiological studies, proton pump inhibitor (PPI) use has been associated with CDI incidence and recurrence. PPIs have also been associated with alterations in the human intestinal microbiota in observational and interventional studies. We evaluated the effects of the PPI omeprazole on the structure of the murine intestinal microbiota and its ability to disrupt colonization resistance to C. difficile. We found omeprazole treatment had minimal impact on the murine fecal microbiota and did not promote C. difficile colonization. Further studies are needed to determine whether other factors contribute to the association between PPIs and CDIs seen in humans or whether aspects of murine physiology may limit its utility to test these types of hypotheses.

## OBSERVATION

Antibiotics have a large impact on the intestinal microbiome and are a primary risk factor for developing Clostridioides difficile infections (CDIs) (1). It is less clear whether other human medications that impact the microbiota also influence C. difficile colonization resistance. The results of multiple epidemiological studies have suggested an association between proton pump inhibitor (PPI) use and incidence or recurrence of CDIs (2–5). There have also been a number of large cohort studies and interventional clinical trials that demonstrated that specific alterations in the intestinal microbiome were associated with PPI use (4, 6). PPI-associated microbiota changes have been attributed to the ability of PPIs to increase stomach acid pH which may promote the survival of oral and pathogenic bacteria (4, 6). In human fecal samples, PPI use results in increases in *Enterococcaceae*, *Lactobacillaceae*, *Micrococcaceae*, *Staphylococcaceae*, and *Streptococcaceae* and decreases in *Ruminococcaceae* (6–9). Several of these taxa have also been associated with C. difficile colonization in humans (10).

Unfortunately, the studies suggesting a link between PPIs and C. difficile were retrospective and did not evaluate changes in the microbiome (2, 3, 5). Thus, it is unclear whether the gastrointestinal microbiome changes associated with PPI use explain the association between PPIs and CDIs. Additionally, epidemiological studies have a limited capacity to address potential confounders and comorbidities in patients who were on PPIs and developed CDIs or recurrent CDIs (2, 5). Here, we evaluated the impact of daily PPI treatment with omeprazole on the murine microbiome and susceptibility to C. difficile colonization in relation to clindamycin, an antibiotic that perturbs the microbiome enough to allow C. difficile to colonize but is mild enough that C. difficile is cleared within 10 days (11).

## 

### Murine fecal microbiomes were minimally affected by omeprazole treatment.

**FIG 1:**
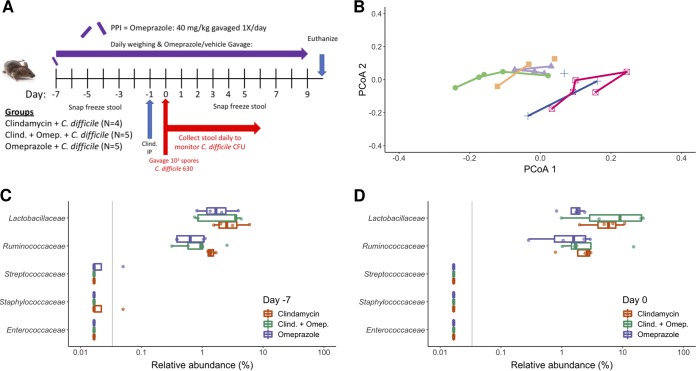
Omeprazole treatment had minimal impact on the murine fecal microbiota. (A) Mouse experiment timeline and logistics. The PPI omeprazole was administered throughout the duration of the experiment. Clindamycin was administered intraperitoneally (IP) 1 day before C. difficile challenge on day 0. Stool samples for 16S rRNA sequencing analysis were collected on the days that are labeled (days −7, −5, −3, −1, 0, 1, 2, 3, 4, 5, 7, and 9). C. difficile CFU in the stool was quantified daily through 6 days postinfection by anaerobic culture. (B) Principal coordinate analysis (PCoA) of Bray-Curtis distances from stool samples of mice in the omeprazole treatment group during the initial 7 days of the experiment. Each color represents stool samples from the same mouse, and lines connect sequentially collected samples. (C and D) Relative abundances of families previously associated with PPI use in humans at the start of the experiment (C) and after 7 days of omeprazole treatment (D). Each circle represents the value for an individual mouse. There were no significant differences across treatment groups for any of the identified families in the sequence data at day −7 (all *P* values  >  0.448) and day 0 (all *P* values  >  0.137), analyzed by Kruskal-Wallis test with a Benjamini-Hochberg correction for multiple comparisons. In panels C and D, the gray vertical line indicates the limit of detection.

To test whether omeprazole treatment alters the microbiome and promotes susceptibility to CDIs, we gavaged mice with 40 mg of omeprazole per kg of body weight for 7 days before C. difficile challenge (Fig. 1A). A principal coordinate analysis (PCoA) of the Bray-Curtis distances over the initial 7 days of treatment revealed that the bacterial communities of omeprazole-treated mice remained relatively unchanged (Fig. 1B). We observed no significant changes in the relative abundance of those taxa previously shown to respond to PPI treatment throughout the course of the 16-day experiment (Fig. 1C and D; see also Fig. S1 in the supplemental material). We also observed no significant changes in relative abundances at the family and genus level over the course of the experiment for the omeprazole-treated mice (all corrected *P* values  >  0.36). These results demonstrated that the omeprazole treatment alone had minimal impact on the murine fecal bacterial community after 7 days of pretreatment.

10.1128/mSphere.00693-19.1 FIG S1 Bacterial families within omeprazole-treated mice fluctuate over time with no overall trend in either direction. (A and B) Relative abundance over time for *Lactobacillaceae* (A) and *Ruminococcaceae* (B), two of the PPI-associated families from human PPI studies across all three treatment groups. Each point represents the relative abundance for an individual mouse stool sample, while the lines represent the mean relative abundances for each treatment group of mice. The gray horizontal lines indicate the limit of detection. Download FIG S1, PDF file, 0.4 MB. Copyright © 2019 Tomkovich et al. 2019 Tomkovich et al. This content is distributed under the terms of the Creative Commons Attribution 4.0 International license.

### Omeprazole treatment did not promote susceptibility to C. difficile infection in mice.

**FIG 2:**
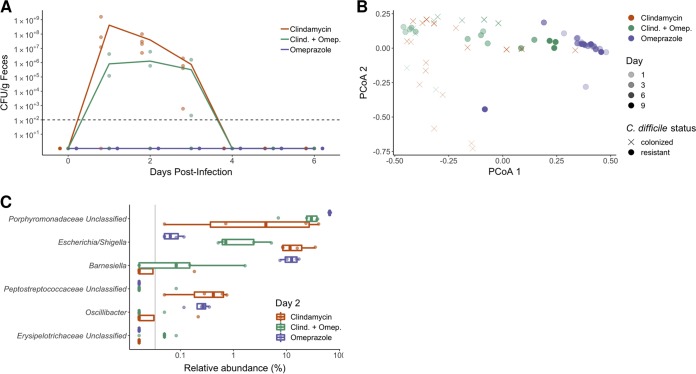
Omeprazole treatment alone does not promote CDIs in mice. (A) C. difficile CFU/gram of stool measured each day after C. difficile challenge for mice treated with clindamycin, clindamycin plus omeprazole, and omeprazole. The lines represent the mean CFU/gram for each treatment group, while points represent CFU/gram for individual mice within each group. The black dashed line indicates the limit of detection. (B) PCoA of Bray-Curtis distances from stool samples collected after antibiotic treatment (last 9 days of the experiment). The hue of the symbol indicates the treatment day. Symbols represent the C. difficile colonization status of the mice measured 2 days postinfection. Circles represent resistant mice (C. difficile was undetectable in stool samples), while × symbols represent mice that were colonized with C. difficile, although all mice cleared C. difficile within 5 days of infection. Omeprazole-treated fecal samples primarily cluster together throughout the experiment. (C) Genera that vary the most across treatment groups for stool samples collected from mice 2 days postinfection. Data were analyzed by Kruskal-Wallis test, and no *P* values were significant after Benjamini-Hochberg correction for multiple comparisons (all *P* values  >  0.092). The gray vertical line indicates the limit of detection.

Next, we examined whether omeprazole treatment altered susceptibility to C. difficile infection in mice. After omeprazole or clindamycin treatment, mice were challenged with 10^3^ C. difficile strain 630 spores. Although C. difficile colonized the clindamycin-treated mice, it did not colonize the omeprazole-treated mice (Fig. 2A). Interestingly, only one cage of mice that received both omeprazole and clindamycin were colonized, while the other cage of mice were resistant (Fig. 2A). The greatest shifts in bacterial communities occurred in the clindamycin-treated mice (Fig. 2B and Fig. S2). Regardless of whether the mice were colonized by C. difficile, all of the mice had cleared C. difficile within 5 days (Fig. 2A), suggesting that omeprazole did not affect the rate of clearance. Our results suggest that omeprazole treatment had no effect on bacterial community resistance to C. difficile colonization in mice. Instead, most of the differences between the three treatment groups appeared to be driven by clindamycin administration (Fig. 2C and Fig. S2). These findings demonstrated that high-dose omeprazole treatment did not promote susceptibility to C. difficile colonization.

10.1128/mSphere.00693-19.2 FIG S2 Microbiota diversity and richness decrease with antibiotic treatment but remain relatively constant with omeprazole treatment. (A and B) Boxplots of the Shannon diversity index values (A) and number of observed OTUs (B) for each group of mice over three time points (days −7, 0, and 9). Each circle represents the value for a stool sample from an individual mouse. Download FIG S2, PDF file, 0.2 MB. Copyright © 2019 Tomkovich et al. 2019 Tomkovich et al. This content is distributed under the terms of the Creative Commons Attribution 4.0 International license.

### Conclusions.

The PPI omeprazole did not meaningfully impact the structure of the gut microbiota and did not promote C. difficile infection in mice. Our findings that omeprazole treatment had minimal impact on the fecal microbiome were comparable to another PPI mouse study that indicated the PPI lansoprazole had more of an effect on the small intestinal microbiota compared to the fecal microbiota (12). The same group demonstrated lansoprazole treatment increased the stomach pH in mice (12), which may improve survival of bacteria passing through the stomach. We did not find significant changes in the relative abundances of the taxa observed to be significantly impacted by PPI use in human studies. However, three of the human-associated taxa were absent or at low abundance in our mice. Interestingly, other groups examining fecal microbiota communities before and after PPI administration to healthy cats and infants with gastroesophageal reflux disease found that PPIs have minimal effects on fecal bacterial community structures, although there were a few significant changes in specific genera (13, 14). One limitation of our study is that there were only four or five mice per group, which may have limited our ability to identify PPI-induced changes in specific bacterial genera. Although our fecal microbiota findings are comparable to what has been shown in another mouse study (12), whether PPI-induced changes in specific bacterial abundances observed in humans play a role in CDIs remains to be determined.

Although several C. difficile mouse model studies have shown that PPIs have an effect on CDIs with or without additional antibiotic treatment (15–17), there were insufficient controls to attribute the effect solely to PPI treatment. One group administered 0.5 mg/kg of the PPI lansoprazole daily for 2 weeks to mice and then challenged with C. difficile demonstrated that PPI treatment alone resulted in detectable C. difficile in stools 1 week after challenge; however, there was detectable C. difficile in mice not treated with antibiotics (15, 16). The other mouse study demonstrated that antibiotic-and-esomeprazole-treated mice developed more severe CDIs than antibiotic-treated mice, but the researchers did not have a group treated with just esomeprazole for comparison (17). We tested the same high 40-mg/kg PPI dose and expanded pretreatment to 7 days before challenge to test the impact of omeprazole treatment alone on our CDI mouse model. Additionally, we have previously demonstrated that mice from our breeding colony are resistant to C. difficile 630 colonization without antibiotic treatment (18), ensuring there was not already partial susceptibility to C. difficile before treatment. The additional controls in our study allowed us to assess the contribution of omeprazole alone to C. difficile susceptibility in mice.

Our study also extended previous work examining PPIs and C. difficile in mice by incorporating the contribution of the intestinal microbiota. We found that omeprazole had no significant impact on bacterial taxa within the murine intestinal microbiota over the 16-day experiment. In contrast to previous work with PPIs (15–17), omeprazole did not alter C. difficile colonization resistance in mice. 16S rRNA sequencing results suggested that *Streptococcus* and *Enterococcus* are rare genera in our C57BL/6 mouse colony. These two genera could be important contributors to the associations between PPIs and CDIs in humans, and could be a contributing factor to our observation that PPI treatment had no effect on C. difficile colonization in our CDI mouse model. While the intestinal microbiomes of both humans and mice are dominated by the *Bacteroidetes* and *Firmicutes* phyla, there are significant differences in the relative abundances of genera that are present and some genera are unique to each mammal (19), differences that may partly explain our results. Gastrointestinal physiological differences, particularly the higher stomach pH in mice (pH 3 to 4) compared to humans (pH 1) (19) could also explain why omeprazole had a limited impact on the murine microbiome. The microbiota and physiological differences between humans and mice may limit the usefulness of employing mouse models to study the impact of PPIs on the microbiota and CDIs.

Beyond microbiome differences, factors such as age, body mass index, comorbidities, and use of other medications in human studies may also be contributing to the association between PPIs and CDI incidence or recurrence. The type of C. difficile strain type could also be an important contributing factor; however, our study was limited in that we tested only C. difficile strain 630 (ribotype 012). This study addressed the impact of PPIs with or without antibiotics on a murine model of CDI and found that PPIs did not promote C. difficile colonization. The epidemiological evidence linking PPIs to CDIs is primarily from observational studies, which makes determining causality and whether other risk factors play a role challenging (20). Future studies are needed to determine whether age, other comorbidities, and bacterial strains that are less common in mice can increase the risk of CDIs or recurrent CDIs when combined with PPI treatment.

### Experimental procedures. (i) Animals.

All mouse experiments were performed with 7- to 12-week-old C57BL/6 male and female mice. Each experimental group of mice was split between two cages with two or three mice housed per cage and male and female mice housed separately. All animal experiments were approved by the University of Michigan Animal Care and Use Committee (IACUC) under protocol number PRO00006983.

### (ii) Drug treatments.

Omeprazole (Sigma-Aldrich) was prepared in a vehicle solution of 40% polyethylene glycol 400 (Sigma-Aldrich) in phosphate-buffered saline (PBS). Omeprazole was prepared from 20 mg/ml frozen aliquots and diluted to 8 mg/ml prior to gavage. All mice received 40 mg of omeprazole per kg of body weight (a dose previously used in mouse experiments [17]) or vehicle solution once per day through the duration of the experiment with treatment starting 7 days before C. difficile challenge (Fig. 1A). Although the omeprazole dose administered to mice is higher than the recommended dose for humans, omeprazole has a shorter half-life in mice compared to humans (21) and lacks an enteric coating (22). One day prior to C. difficile challenge, two groups of mice received an intraperitoneal injection of 10 mg/kg clindamycin or sterile saline vehicle (11). All drugs were filter sterilized through a 0.22-μm syringe filter before administration to animals.

### (iii) C. difficile infection model.

Mice were challenged with C. difficile strain 630 7 days after the start of omeprazole treatment and 1 day after clindamycin treatment. Mice were challenged with 10^3^ spores in ultrapure distilled water as described previously (11). Stool samples were collected for 16S rRNA sequencing or C. difficile CFU quantification throughout the duration of the experiments at the indicated time points (Fig. 1A). Samples for 16S rRNA sequencing were flash frozen in liquid nitrogen and stored at –80°C until DNA extraction, while samples for CFU quantification were transferred into an anaerobic chamber and serially diluted in PBS. Diluted samples were plated on TCCFA (taurocholate- cycloserine-cefoxitin-fructose agar) plates and incubated at 37°C for 24 h under anaerobic conditions to quantify C. difficile CFU.

### (iv) 16S rRNA gene sequencing.

DNA for 16S rRNA gene sequencing was extracted from 10 to 50 mg fecal pellet from each mouse using the DNeasy Powersoil HTP 96 kit (Qiagen) and an EpMotion 5075 automated pipetting system (Eppendorf). The 16S rRNA sequencing library was prepared as described previously (23). In brief, the ZymoBIOMICS microbial community DNA standard (Zymo, CA, USA) was used as a mock community (24), and water was used as a negative control. The V4 hypervariable region of the 16S rRNA gene was amplified with Accuprime Pfx DNA polymerase (Thermo Fisher Scientific) using previously described custom barcoded primers (23). The 16S rRNA amplicon library was sequenced with the MiSeq system (Illumina). Amplicons were cleaned up and normalized with the SequalPrep normalization plate kit (Thermo Fisher Scientific), and pooled amplicons were quantified with the KAPA library quantification kit (KAPA Biosystems).

### (v) 16S rRNA gene sequence analysis.

mothur (v1.40.5) was used for all sequence processing steps (25) using a previously published protocol (23). In brief, forward and reverse reads for each sample were combined, and low-quality sequences and chimeras were removed. Duplicate sequences were merged, before taxonomy assignment using a modified version (v16) of the Ribosomal Database Project reference database (v11.5) with an 80% cutoff. Operational taxonomic units (OTUs) were assigned with the opticlust clustering algorithm using a 97% similarity threshold. To adjust for uneven sequencing across samples, all samples were rarefied to 3,000 sequences 1,000 times. PCoAs were generated based on Bray-Curtis distance. R (v.3.5.1) was used to generate figures and perform statistical analysis.

### (vi) Statistical analysis.

To test for differences in relative abundances in families and genera across our three different treatment groups at different time points (clindamycin, clindamycin plus omeprazole, and omeprazole on days −7, 0, 2, and 9) or within the omeprazole treatment group across three time points (days −7, 0, and 9), we used a Kruskal-Wallis test with a Benjamini-Hochberg correction for multiple comparisons.

### Code availability.

The code for all sequence processing and analysis steps as well as a Rmarkdown version of this manuscript is available at https://github.com/SchlossLab/Tomkovich_PPI_mSphere_2019.

### Data availability.

The 16S rRNA sequencing data have been deposited in the NCBI Sequence Read Archive (accession no. PRJNA554866).
