# Supplementary figures and images for: The Proton Pump Inhibitor Omeprazole Does Not Promote Clostridioides difficile Colonization in a Murine Model
Source: mSphere. 2019 Nov 20;4(6):e00693-19. doi: 10.1128/mSphere.00693-19 (PMC6887860; doi:10.1128/mSphere.00693-19)

**A**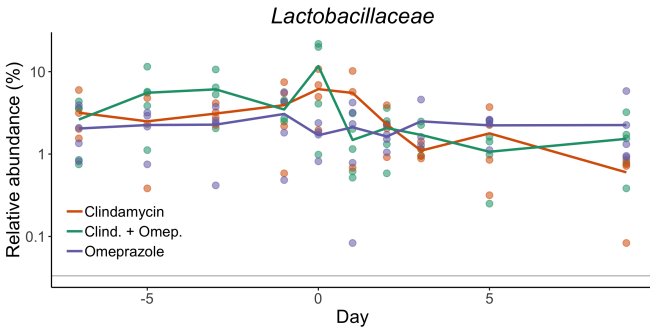**B**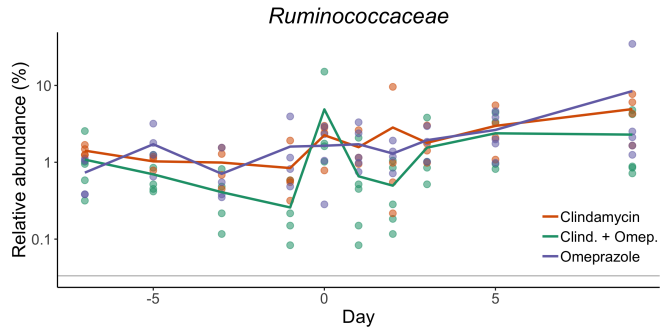

Supplement: FIG S1 [file mSphere.00693-19-sf001.pdf]

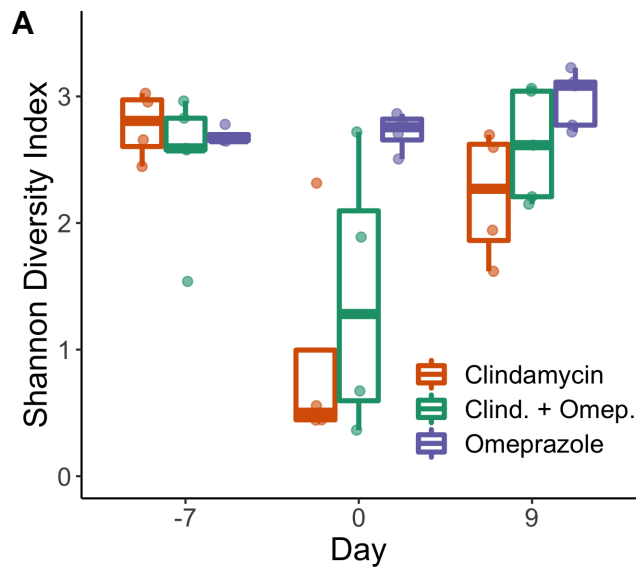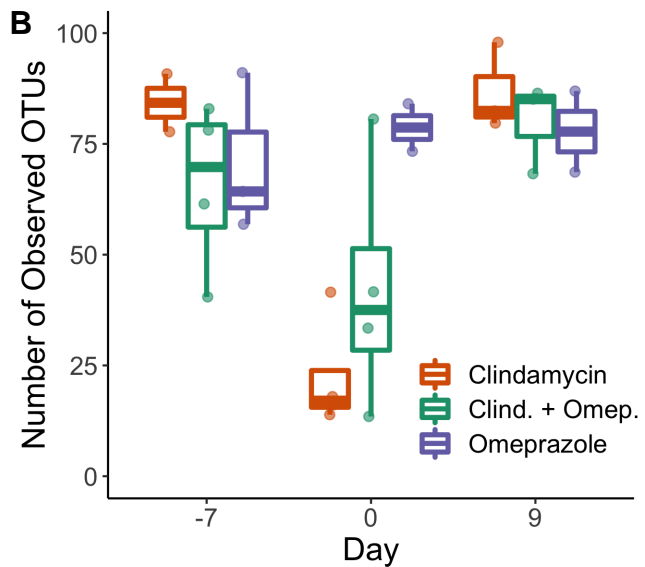

Supplement: FIG S2 [file mSphere.00693-19-sf002.pdf]
